# Routine DTP Vaccination Coverage and Herd Immunity Against Pertussis in 2024 Did Not Recover to Pre-COVID-19 Levels Globally and in WHO Regions

**DOI:** 10.3390/vaccines14030264

**Published:** 2026-03-13

**Authors:** Pedro Plans-Rubió

**Affiliations:** College of Physicians of Barcelona, 08017 Barcelona, Spain; pedro.plans@yahoo.es

**Keywords:** health indicators, vaccine coverage, pertussis vaccines, zero-dose, three-dose coverage, herd immunity, pertussis prevention, pandemic impact, WHO regions, Immunization Agenda 2030

## Abstract

**Objectives**: The study’s objective was to assess ten diphtheria–tetanus–pertussis (DTP) vaccination program indicators globally and in World Health Organization (WHO) regions in 2024, and compare the values in 2024 and 2019. **Methods**: Global and regional values for routine DTP vaccination performance indicators were assessed in 2024. Means and percentages in 2024 and 2019 were compared using the *t*-test and Chi-square test, respectively, considering *p* < 0.05 as statistically significant. High-priority countries for DTP vaccination coverage increase were identified in each WHO region based on the indicators assessed in this study. **Results**: The global mean vaccination coverage for DTP1, DTP3 and three DTP doses were 90.7%, 86.6% and 72.8%, respectively, in 2024. Eight of the ten indicators assessed in this study worsened and two improved globally from 2019 to 2024. The differences between 2019 and 2024 were statistically significant for the three-dose DTP coverage decrease in the European WHO region (88.1% vs. 82.5%, *p* < 0.05), and the decrease in the global percentage of countries with ≥90% three-dose coverage (34% vs. 21%, OR = 0.52, 95% CI: 0.33–0.81, *p* < 0.005). This study identified 27 (13.8%) high-priority countries for DTP vaccination coverage increase due to DTP1 coverage lower than 80%; 47 (24.1%) countries due to DTP3 coverage lower than 80%; and 48 (24.6%) countries due to three-dose coverage lower than 60%. **Conclusions**: Global and regional DTP vaccination performance indicators in 2024 did not recover to pre-pandemic levels, although the differences between 2024 and 2019 were statistically significant only for two regional indicators.

## 1. Introduction

Pertussis or whooping cough is an endemic infectious disease caused by *Bordetella pertussis*. Pertussis infections can generate outbreaks and epidemics affecting individuals of all ages. However, the incidence of pertussis and the risk of pertussis complications are greater among children aged less than one year and adolescents than among adults [1,2]. A study quantifying the burden of pertussis in 2021 among individuals under 20 years old across regions with low and high socio-demographic index (SDI), found that infants under 1 year old and children aged 1–9 years had a higher pertussis incidence and mortality than individuals aged 10–20 years, and that low-SDI regions had higher pertussis incidence and mortality than high-SDI regions [3].

Despite the availability of pertussis vaccines since 1960, pertussis cases and outbreaks have occurred every year worldwide and pertussis resurgence has been reported in different countries in the past two decades [4,5]. The number of pertussis cases reported worldwide ranged from I49,000 to 174,000 prior to the COVID-19 pandemic (2015–2020) and decreased to less than 70,000 during the pandemic [6]. However, pertussis resurged since 2022 and 941,565 cases were reported worldwide in 2024, with 591,193 in the Western Pacific region and 298,595 in the European region [6].

In 2024, the number of pertussis cases reported was 476% greater than that registered in 2023. The global incidence of pertussis, which ranged from 4.9 to 9 per million population during 2020–2022, increased to 137 per million population in 2024 [6]. In 2024, pertussis incidence was 324 and 307 per million population in the European and Western Pacific regions, respectively [6].

Several factors have been proposed to explain pertussis resurgence, including insufficient vaccination coverage, insufficient anti-pertussis herd immunity, waning of vaccine-induced immunity over time, and lower vaccine effectiveness due to *Bordetella pertussis* changes after widespread vaccination [7,8,9,10].

Routine pertussis vaccination is the key intervention to achieve prevention and control [11]. The Immunization Agenda 2030 (IA2030) endorsed by the 73rd World Health Assembly proposed an ambitious strategy for vaccines and immunization for the 2021–2030 decade [12]. The routine schedule for administering diphtheria–tetanus–pertussis-containing (DTP) vaccines to children is a three-dose series at 2 months, 4 months, and 6–12 months, followed by two booster doses at 15–18 months and 4–6 years [10,11,12,13]. However, routine DTP vaccination schedules can vary by country, and the two booster doses can be given between the ages of 12 months and 16 years [10,11,12,13]. Vaccination programs can use pertussis-containing vaccines including acellular or whole-cell pertussis antigens. Acellular (aP) and whole-cell (wP) pertussis vaccines are used worldwide to prevent pertussis in infants, adolescents and adults [10,11,12,13]. In 2024, 45% of countries had included the aP vaccine in routine vaccination programs, while 55% of them used wP vaccines [14]. The wP vaccine remains the vaccine used in most countries of Africa and South and West Asia [14].

The pertussis prevention strategy based on routine vaccination requires achieving high percentages of coverage and high levels of pertussis vaccination effectiveness to prevent community transmission [10]. The Immunization Agenda 2030 proposed to achieve 90% vaccination coverage for the first and third doses of DTP-containing vaccines by 2030 at the national and regional levels [15].

A prior study found global mean vaccination coverages with three, two, one and zero doses of DTP vaccine in 2019 of 77%, 18%, 4.3% and 0.7%, respectively [10]. In addition, three-dose DTP vaccination coverage was greater than 90% in only 34% of countries worldwide [10]. The objectives of this study were: (1) to assess the vaccination coverage for three, two, one and zero doses of the DTP vaccine in all countries; (2) to assess the mean vaccination coverage for DTP1 and DTP3 vaccines, and three, two, one and zero doses of the DTP vaccine in different WHO regions in 2024; (3) to assess the prevalence of vaccine-induced pertussis protection in all counties and WHO regions in 2024; (4) to assess variations from 2019 to 2024 in DTP vaccination coverage and anti-pertussis herd immunity indicators in different WHO regions; and (5) to assess whether zero-dose vaccination coverage indicators and the number of zero-dose children are on track to achieve the Immunization Agenda 2030 objective.

## 2. Methods

### 2.1. Mean Vaccination Coverage with DTP1 and DTP3 Vaccines, and Mean Three-Dose and Zero-Dose DTP Vaccination Coverage in the WHO Regions in 2024

Mean percentages of vaccination coverage with the first (DTP1) and third (DTP3) dose of the DTP-containing vaccine, and mean three-dose and zero-dose DTP vaccination coverage in one-year-old children (target population) were determined globally and in each WHO region using the information about the DTP1 and DTP3 vaccination coverage in different countries. WHO and UNICEF produce country-specific estimates for routine DTP1 and DTP3 vaccination coverage by individually reviewing each country’s data about annual administrative-based official coverage and survey reports [16,17,18,19]. Administrative DTP1 and DTP3 vaccination coverage is defined as the number of DTP1 and DTP3 doses administered to targeted children divided by the estimated target population [19].

In this study, two indicators of DTP vaccination program performance were assessed: (1) three-dose DTP coverage and (2) zero-dose DTP coverage among one-year-old children (target population). The three-dose and zero-dose DTP vaccination coverage among one-year-old children can be considered indicators of vaccination program performance in different countries and WHO regions, as they show the percentage of one-year-old children who have completed the DTP vaccination and the percentage of one-year-old children who have not received any DTP dose, respectively.

Two methods were applied to assess the three-dose and zero-dose DTP vaccination coverage in different countries and WHO regions: (1) WHO approach and (2) conservative approach. Based on the WHO approach, three-dose coverage was equal to DTP3 coverage, and zero-dose coverage was calculated from 100—DTP1 coverage [18]. The WHO approach assumed that children vaccinated with the DTP3 vaccine had received the DTP1 and DTP2 vaccines previously, and that children unvaccinated with the DTP1 vaccine did not receive other DTP doses [18].

Based on a conservative approach, the three-dose coverage was determined from DTP1 × DTP2 × DTP3, and the zero-dose coverage was determined by assuming that children unvaccinated with the DTP1 vaccine could have received the DTP2 and/or DTP3 vaccines. The distribution of DTP doses among one-year-old children assumed in the conservative approach is more equitable than that assumed by WHO-UNICEF for two reasons: (1) the main concern regarding routine DTP vaccination is focused on zero-dose children and zero-dose coverage; and (2) percentages of zero-dose vaccination coverage are lower using the conservative approach than using the WHO-UNICEF method. The method to assess the three-dose and zero-dose coverage based on the conservative approach is explained in detail in a prior article [10].

### 2.2. Anti-Pertussis Herd Immunity Levels in the Target Vaccination Population in WHO Regions in 2024

Anti-pertussis herd immunity levels in different WHO regions were determined using the country-based mean prevalence of vaccine-induced pertussis protection in children aged one year old. This prevalence was determined in each country from the vaccination coverage with one, two and three doses of DTP vaccine and values of effectiveness in preventing pertussis cases of 84%, 77%, and 59% with one, two and three doses of vaccine, respectively [10,20,21,22].

Herd immunity against *Bordetella pertussis* was considered to be established in each country and WHO region when the prevalence of pertussis protection was higher than 90%, 90.9%, 91.7%, 92.3%, 92.9%, 93.3%, 93.8%, 94.1% and 94.4% for basic reproduction numbers (R_o_) of 10, 11, 12, 13, 14, 15, 16, 17 and 18, respectively [10,23]. The percentage of countries with sufficient herd immunity against *Bordetella pertussis* with Ro values from 10 to 18 was determined in each WHO region.

The basic reproduction number is a measure of the transmissibility of *Bordetella perussis* in a population, defined as the average number of secondary pertussis cases produced per infective case in a totally susceptible population [23]. It depends on the characteristics of the infectious agent (e.g., infectivity and duration of infectiousness) and of the population (e.g., population density and social mixing patterns) [23].

### 2.3. Assessment of Whether Zero-Dose DTP Vaccination Indicators in 2024 Were on Track to Achieve the Immunization Agenda 2030 Objective

The tracks required to achieve a worldwide and regional 50% reduction from 2019 to 2030 were assessed for the following vaccination performance indicators: (1) number of zero-dose children [17,18]; (2) mean zero-dose coverage (100 − DTP1); and (3) mean zero-dose coverage derived from the coverage for one, two and three doses of DTP vaccine (conservative approach) [10].

For the number of zero-dose children, the 2019–2030 track was determined by taking into account the zero-dose children in 2019 (12.86 million) [17,18] and its 50% reduction in 2030 (9.0 million). The estimated number of zero-dose children in 2024 was compared with the number required to achieve the IA2030 objective.

For the mean zero-dose coverage (DTP1-based), the 2019–2030 track was determined by taking into account the mean zero-dose coverage in 2019 (7.4%) [10] and its 50% reduction in 2030 (3.7%). The mean zero-dose coverage in 2024 was compared with the coverage required to achieve the IA2030 objective.

For the mean zero-dose DTP coverage derived from one-, two- and three-dose coverage, the 2019–2030 track was determined by taking into account the mean zero-dose coverage in 2019 (0.7%) [10] and its 50% reduction in 2030 (0.35%). The mean zero-dose coverage in 2024 was compared with the coverage required to achieve the IA2030 objective.

### 2.4. High-Priority Countries for Routine DTP Vaccination Coverage Increase 

In this study, high-priority countries for DTP vaccination coverage increase were identified in each WHO region using the following criteria:

• Countries with zero-dose DTP coverage greater than the regional mean zero-dose coverage.
• Countries with three-dose DTP coverage lower than 60%.
• Countries with DTP1 coverage lower than 80%.
• Countries with DTP3 coverage lower than 80%.

### 2.5. Statistical Analysis

Microsoft Excel (Microsoft Corporation, Redmond, WA, USA) was used to calculate: (1) the vaccination coverage with three, two, one and zero DTP doses in each country and mean regional coverages in 2024; (2) the prevalence of pertussis protection in each country and mean regional prevalences in 2024; and (3) the variation from 2019 to 2024 for vaccination coverage and anti-pertussis herd immunity indicators in WHO regions.

Microsoft Excel (Microsoft Corporation, Redmond, WA, USA) was used to assess the establishment of anti-pertussis herd immunity in different countries and WHO regions in 2024, and to assess whether zero-dose vaccination coverage indicators were on track to achieve the Immunization Agenda 2030 objective.

Means and their 95% confidence intervals were determined for quantitative DTP vaccination indicators. Percentages and their 95% confidence intervals were determined for qualitative DTP vaccination indicators. Student’s *t*-test was used to compare means in 2019 and 2024, considering *p* < 0.05 as statistically significant. The Chi-square test (Fisher’s exact test when necessary) and odds ratios were used to compare percentages in 2019 and 2024, considering *p* < 0.05 as statistically significant. All statistical analyses were performed using the IBM-SPSS program (Version 18, IBM-SPSS, Chicago, IL, USA).

## 3. Results

### 3.1. Mean DTP1 and DTP3 Vaccination Coverage in 2024

This study found global means for DTP1 and DTP3 vaccination coverage of 90.7%, 86.6%, respectively, in 2024 (Table 1). The mean DTP1 coverage was greater than 90% in the European, South-East Asia and Western Pacific regions, while the mean DTP3 coverage was greater than 90% only in the European and South-East Asia regions (Table 1).

The global mean zero-dose coverage determined from the DTP1 coverage was 9.3%, and it ranged from 5.2% in the European region to 14.5% in the African region (Table 1).

### 3.2. Mean Percentages of Vaccination Coverage with Three, Two, One and Zero Doses of DTP Vaccine in 2024 (Conservative Approach)

This study found global means for three-dose and zero-dose DTP coverage of 72.8% and 0.8%, respectively, in 2024 (Table 1). The three-dose coverage ranged from 82.5% in the European region to 59.5% in the African region; the zero-dose coverage ranged from 1.2% in the African region to 0.2% in the European region (Table 1).

The global means for two-dose and one-dose coverage were 21.1% and 5.3%, respectively, in 2024 (Table 1). The mean two-dose coverage ranged from 30.4% in the African region to 15.3% in the European region, while the one-dose coverage ranged from 8.9% in the African region to 2% in the European region (Table 1).

The three-dose DTP coverage was ≥95% and ≥90% in 12.3% and 21% of the countries worldwide, respectively (Table 1). The percentage of countries with three-dose DTP coverage ≥ 90% was 18–37% in the Americas, European, South-East Asia and Western Pacific regions, while it was lower than 10% in the African and Eastern Mediterranean regions (Table 1).

### 3.3. Anti-Pertussis Herd Immunity Levels in 2024

This study found a global mean prevalence of vaccine-induced pertussis protection among one-year-old children (target vaccination population) of 80.5% (Table 1). The prevalence of pertussis protection ranged from 82.3% in the European region to 78.6% in the African region (Table 1).

In 2024, anti-pertussis herd immunity levels generated by routine DTP vaccination programs were not sufficient to block pertussis transmission among one-year-old children in all WHO regions, as the prevalence of vaccine-induced protection was lower than the prevalence of 90% required against pertussis with transmissibility in terms of a basic reproduction number of 10 in all WHO regions (Table 1).

The herd immunity analysis did not change considering the 95% confidence interval for the mean prevalence of vaccine-induced immune protection, as the highest values of the 95% confidence intervals were lower than 90% in all WHO regions (Table 1).

### 3.4. Variation in DTP Vaccination Coverage and Anti-Pertussis Herd Immunity Indicators from 2019 to 2024

Eight of the DTP vaccination program indicators assessed in this study worsened and two improved from 2019 to 2024. The following indicators worsened worldwide from 2019 to 2024: mean DTP1 coverage (−2%), mean DTP3 coverage (−1.8%), mean DTP1-based zero-dose coverage (25.4%); mean three-dose coverage (−5.3%), mean zero-dose coverage (14.3%) (conservative approach), percentage of countries with three-dose coverage ≥ 95% (−31.7%), percentage of countries with three-dose coverage ≥ 90% (−38.2%), and mean prevalence of children aged one year with vaccine-induced pertussis protection (−0.7%) (Table 2). By contrast, the indicators two-dose and one-dose DTP coverage improved by 17.2% and 23.3%, respectively, from 2019 to 2024 (Table 2). Supplementary Table S1 presents the values for indicators of DTP vaccination in 2019.

The differences between 2019 and 2024 were statistically significant for the following global and regional indicators: decrease in the global three-dose coverage in the European region (88.1% in 2019 vs. 82.5% in 2024, *p* < 0.05); increase in the global two-dose coverage (18% in 2019 vs. 21.1% in 2024, *p* < 0.05); increase in the two-dose coverage in the European region (11.1% in 2019 vs. 15.3% in 2024, *p* < 0.05); and decrease in the global percentage of countries with ≥90% three-dose coverage (34% vs. 21%, OR = 0.52, 95% CI: 0.33–0.81, *p* < 0.005) (Table 2).

This study found that the mean DTP1 and mean DTP3 vaccination coverage decreased from 2019 to 2024 in all WHO regions, except in the Western Pacific region (Table 2). However, the differences for DTP1 and DTP3 coverage between 2019 and 2024 were not statistically significant.

The mean zero-dose coverage (100 − DTP1 coverage) increased from 2019 to 2024 in all WHO regions. The South-East Asia region had the greatest (57.8%) and the African region had the lowest (6.7%) zero-dose coverage increase from 2019 to 2024 (Table 2). However, the differences between 2019 and 2024 were not statistically significant for all WHO regions.

The mean three-dose DTP vaccination coverage (conservative approach) decreased from 2019 to 2024 in all WHO regions, but differences between 2019 and 2024 were only statistically significant for the European region (*p* < 0.05) (Table 2).

The mean zero-dose coverage (conservative approach) decreased from 2019 to 2024 in the African and Western Pacific regions, did not vary in the European and Western Pacific regions, and increased in the Americas, Eastern Mediterranean, European, and South-East Asia regions (Table 2). However, the differences between 2019 and 2024 were not statistically significant.

The percentage of countries with three-dose DTP coverage ≥ 95% increased or did not vary from 2019 to 2024 in the African, Americas and European regions, and decreased in the other regions (Table 2). The percentage of countries with three-dose coverage ≥ 90% increased or did not vary from 2019 to 2024 in the African and Western Pacific regions, and decreased in the other regions (Table 2). However, the differences between 2019 and 2024 were not statistically significant.

The mean prevalence of one-year-old children with vaccine-induced pertussis protection and anti-pertussis herd immunity levels did not vary significantly from 2019 to 2024 in all WHO regions (Table 2).

### 3.5. Assessment of Whether Zero-Dose DTP Vaccination Indicators in 2024 Were on Track to Achieve the IA2030 Objective by 2030

**Figure 1:**
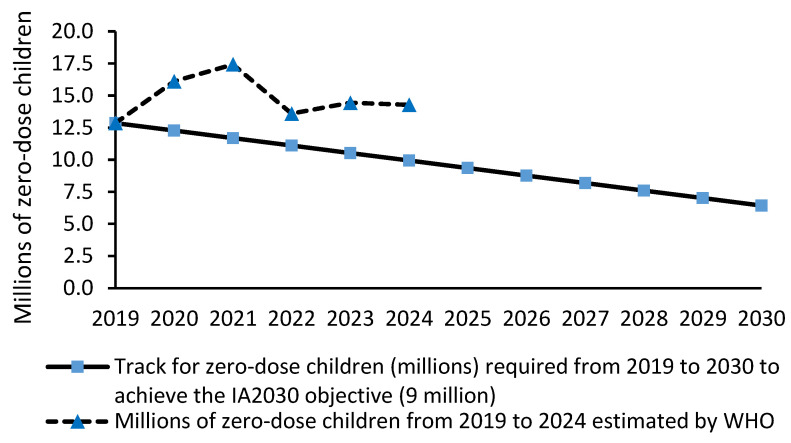
Track for zero-dose DTP children (millions) required from 2019 to 2030 to achieve the IA2030 objective (6.43 million), and number of zero-dose children worldwide from 2019 to 2024 estimated by WHO.

**Figure 2:**
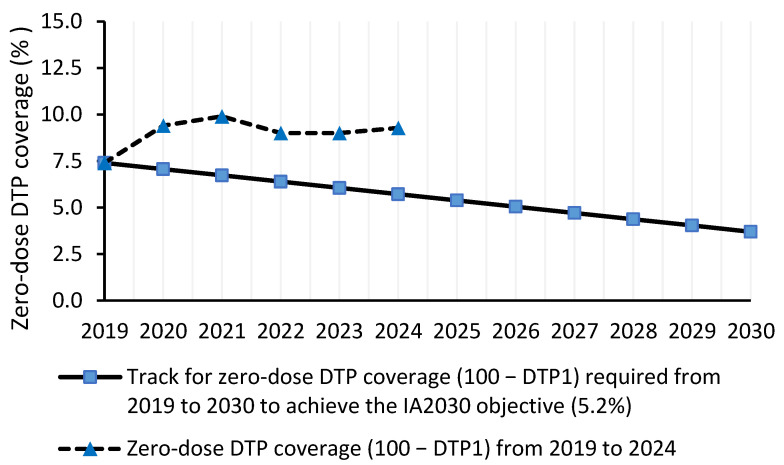
Track for zero-dose DTP vaccination coverage (determined from 100 − DTP1 coverage) required from 2019 to 2030 to achieve the IA2030 objective (3.7%), and zero-dose DTP coverage from 2019 to 2024 determined in this study.

**Figure 3:**
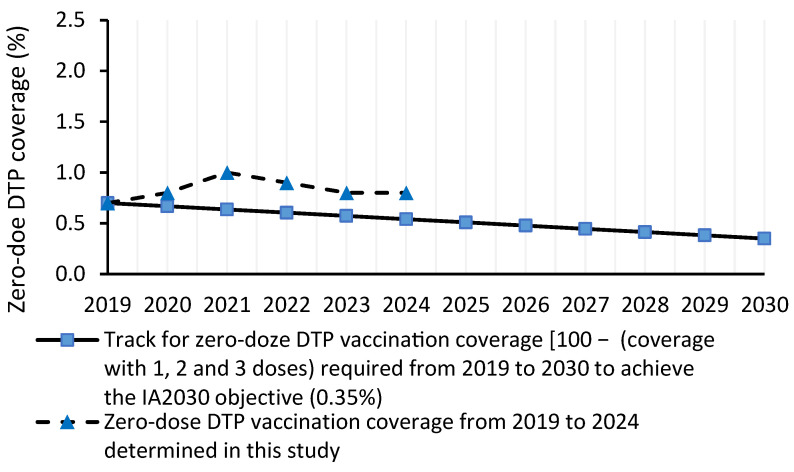
Track for zero-dose DTP vaccination coverage [determined from 100 − (one-dose coverage + two-dose coverage + three-dose coverage)] required from 2019 to 2030 to achieve the IA 2030 objective (0.35%), and zero-dose DTP coverage from 2019 to 2024 determined in this study.

The three zero-dose DTP coverage indicators assessed in this study were not on track to achieve the IA2030 objective of reducing the 2019 levels by 50% in 2030 (Figure 1, Figure 2 and Figure 3). In fact, in 2024, the number of zero-dose children, mean DTP1-based zero-dose coverage and mean zero-dose coverage were 43.9%, 62.2% and 48.1% greater, respectively, than the values required in 2024 to achieve the IA2030 objective.

The number of zero-dose children in 2024 (14.3 million) [16] was not on track to achieve 6.43 million by 2030 (Figure 1). The number of zero-dose children required in 2024 was 9.94 million (Figure 1).

The mean zero-dose DTP1-based coverage in 2024 (9.28%) was not on track to achieve a coverage of 3.7% by 2030 (Figure 2). The zero-dose DTP1-based coverage required in 2024 was 5.72% (Figure 2).

The mean zero-dose coverage in 2024 (0.8%) (conservative approach) was not on track to achieve a coverage of 0.35% by 2030 (Figure 3). The zero-dose coverage required in 2024 was 0.54% (Figure 3).

### 3.6. High-Priority Countries for Routine DTP Vaccination Coverage Increase

This study identified 37 (19%) countries with high-priority for routine DTP vaccination coverage increase due to zero-dose coverage greater than the regional mean; forty-eight (24.6%) countries due to three-dose coverage lower than 60%; twenty-seven (13.8%) countries due to DTP1 coverage being lower than 80%; and 47 (24.1%) countries due to DTP3 coverage lower than 80% (Table 3).

The European and South-East Asia regions had the lowest percentages of high-priority countries among WHO regions, with less than 20% high-priority countries for each one of the four criteria (Table 3). The African region had the greatest percentages of high-priority countries for DTP vaccination coverage increase among WHO regions, with more than 27% high-priority countries for each one of the four criteria (Table 3).

Supplementary Table S2 presents the list of high-priority countries for routine DTP vaccination coverage increase identified in each WHO region, based on the four criteria considered in this study.

## 4. Discussion

This study found a global mean DTP1 vaccination coverage slightly greater than 90%, while the mean DTP3 and three-dose DTP vaccination coverage were lower than 90% worldwide and in all WHO regions. Furthermore, vaccine-induced anti-pertussis herd immunity levels in children aged one year were lower than those necessary to block *Bordetella pertussis* transmission worldwide and in all WHO regions in 2024.

In 2024, the European, South-East Asia and Western Pacific regions had indicators of DTP vaccination program performance better than the other WHO regions, with percentages of DTP1 and DTP3 coverage greater than 90%, three-dose DTP coverage greater than 77%, and prevalence of one-year-old children with vaccine-induced pertussis protection greater than 80%. By contrast, the African region had lower DTP vaccination performance than the other WHO regions, with percentages of DTP1 and DTP3 coverage lower than 90%, three-dose DTP coverage lower than 60%, and prevalence of one-year-old children with vaccine-induced pertussis protection lower than 80%.

This study found that eight of the ten routine DTP indicators assessed in this study worsened globally and in WHO regions from 2029 to 2024, including the mean DTP1 coverage, mean DTP3 coverage, three-dose coverage, zero-dose coverage, percentage of countries with three-dose coverage ≥ 95%, percentage of countries with three-dose coverage ≥ 90%, percentage of countries where all children aged one year had received at least one dose of DTP vaccine, and mean prevalence of children aged one year with vaccine-induced pertussis protection. However, the differences between 2019 and 2024 were statistically significant only for the three-dose DTP coverage decrease in the European region and the global decrease in the percentage of countries with ≥90% three-dose coverage.

This study found that routine DTP vaccination programs could not generate sufficient herd immunity to block *Bordetella pertussis* transmission worldwide and in different WHO regions, as the estimated prevalence of one-year-old children with vaccine-induced pertussis protection was lower than lower than 90% worldwide and in all WHO regions. However, the establishment of herd immunity among one-year-old children in each country depends on the prevalence of vaccine-induced protection, and factors that can increase or reduce pertussis transmissibility, including the population density, use of protective measures and social mixing patterns [23]. Herd immunity levels were determined by assuming a homogeneous mixing of the population, and the potential effect of factors that can increase or reduce pertussis transmissibility have not been assessed in this study.

The insufficient levels for routine DTP vaccination coverage and anti-pertussis herd immunity are two factors explaining pertussis resurgence. Other explanatory factors include the type of vaccine, waning pertussis immunity, lower effectiveness of DTP-containing vaccines due to *Bordetella pertussis* mutations, and a lack of or insufficient vaccination coverage with booster doses among preschool children, adolescents and adults [7,8,9,10,21,24,25,26,27,28]. Recent studies have found that *Bordetella pertussis* without the pertactin and pertussis toxin vaccine antigens could also favor pertussis resurgence in 2024. Several studies have found that emergent *Bordetella pertussis* strains with non-vaccine antigens could be associated with lower levels of acellular pertussis vaccine effectiveness [8,9,24,26,27]. A study carried out in the United States found an association between the switch from whole-cell to acellular pertussis vaccines and pertussis incidence registered from 1992 to 2024, one that was statistically significant after adjusting for vaccination coverage [24]. However, a direct comparison between pertussis incidence in countries using acellular (aP) vaccines and countries using whole-cell (wP) vaccines is difficult due to differences in pertussis surveillance activities, pertussis case management, the definition of pertussis, and laboratory detection methods [4,29,30,31,32,33]. In fact, the worldwide impact of emerging *Bordetella pertussis* without vaccine antigens on the effectiveness of DTP-containing vaccines and herd immunity levels has not been assessed.

This study’s finding that global and regional DTP vaccination indicators did not recover completely to pre-pandemic levels in 2024 could be explained by the following factors: (1) persistence of the COVID-19 pandemic’s effects on routine vaccination programs, (2) vaccine hesitancy, and (3) insufficient health and economic resources for routine vaccination programs. The COVID-19 pandemic disrupted routine DTP vaccination programs due to lockdowns, logistical problems, and the reallocation of health and economic resources to COVID-19 vaccination, detection and management [34,35]. The WHO-UNICEF and studies focused on countries and WHO regions found that DTP vaccine coverage dropped during the pandemic period and that COVID-19-associated vaccination disruptions varied across countries and WHO regions [34,35,36,37,38,39].

The COVID-19 pandemic’s impact on routine DTP vaccination could still have been in effect in many countries in 2024 if the health and economic resources necessary to implement routine DTP vaccination activities did not recover their pre-COVID-19 levels. Parents’ hesitancy to vaccinate their children with DTP-containing vaccines could be another factor contributing to the lower DTP vaccination performance. Parental vaccine hesitancy has been associated with 25% lower vaccination coverage among children aged 19–35 months [40]. Such hesitancy includes concerns for vaccine safety and effectiveness [41], a lack of information [42], and low trust in vaccines and health services [43]. A study comparing parental attitudes and behaviors towards childhood vaccination with DTP-containing vaccines in the United Kingdom and Israel before and after the COVID-19 pandemic found that 5.1% and 6.6% of parents reported a shift towards not-vaccinating their child after the pandemic vaccines in their countries, respectively [44]. The study found that after the COVID-19 pandemic, concerns regarding potential side effects were greater and trust in vaccines was lower than before the pandemic [44].

The results found in this study show that it is necessary to develop a more ambitious pertussis vaccination strategy than that based only on routine vaccination of children, one with the objective of ensuring individual protection among one-year-old children and generating sufficient anti-pertussis herd immunity to prevent *Bordetella pertussis* transmission in the population [10,45]. This strategy must include the following components: (1) ≥90% routine three-dose DTP vaccination coverage in children aged one year; (2) vaccination of adolescents and adults every 10 years with booster doses of pertussis vaccine [45,46]; and (3) vaccination of pregnant women with a single dose of Tdap or Tdap-IPV vaccines between 27 and 36 weeks of gestation and of unvaccinated women who have recently given birth [47,48]. In the European region, pertussis vaccination in adolescents and adults differs from country to country, with all countries recommending booster doses in adolescents and 12 countries recommending adult vaccination with 1–2 booster doses or booster doses every 10 years after the last dose [46]. Supplementary DTP vaccination activities can be developed to increase pertussis immunity in countries with very low vaccination coverage, especially in the African region [18]. However, intensive supplementary vaccination activities should not be undergone over a long period of time, because they demand significant resources and can disrupt routine vaccination activities [49,50].

The study finding that the three zero-dose indicators in 2024 were not on track to achieve the IA2030 objectives showed that increasing the routine DTP coverage must have the highest priority in the pertussis prevention strategy for two main reasons: (1) infants aged less than one year and children aged 1–9 years have the highest pertussis incidence and mortality rates [3,4]; (2) to reach routine ≥ 90% DTP vaccination coverage is a critical step to achieve and maintain sufficient herd immunity among one-year-old children and in other age groups.

Different criteria can be used to decide which countries in each WHO region must be high priority for routine DTP vaccination coverage increase. This study identified 37 high-priority countries for DTP vaccination coverage based on the zero-dose coverage, 48 countries based on the three-dose coverage < 80%, 27 countries based on the DTP1 coverage < 80% and 47 countries based on the DTP3 coverage < 80% (Supplementary Table S2). The WHO and UNICEF gave high priority to increase the DTP coverage to (1) the 10 countries with the highest number of unvaccinated one-year-old children (Nigeria, India, Sudan, Democratic Republic of Congo, Ethiopia, Indonesia, Yemen, Angola and Pakistan); and (2) the 10 countries with the lowest DTP1 coverage (Sudan, Angola, Afghanistan, Azerbaijan, Bolivia, Gabon, Papua New Guinea, and Democratic Republic of Congo) [17]. The results obtained in this study showed that it is necessary to increase routine DTP vaccination coverage in the African region and in countries with high zero-dose coverage and low levels of DTP1, DTP3 and three-dose of vaccination coverage (Supplementary Table S1). In all WHO regions, the objective must be to reach >80% coverage with DTP1 and DTP3 vaccines and >60% three-dose DTP coverage.

The analysis carried out in this study has four limitations. Firstly, the analysis was carried out using the information provided by different countries on DTP1 and DTP3 vaccines distributed and administered to the target population to the WHO-UNICEF immunization information system. However, the routine DTP1 and DTP3 vaccination coverage reported by the WHO-UNICEF immunization information system is periodically validated by the WHO [18,19,51]. Secondly, the vaccination coverage for three, two and one doses of the DTP vaccine were determined by estimating the DTP2 vaccination coverage from the mean DTP1 and DTP3 coverage. However, this method can be considered adequate because the WHO-UNICEF immunization information system does not provide data for the DTP2 vaccine. Thirdly, anti-pertussis herd immunity levels in different countries and WHO regions were assessed by assuming vaccine effectiveness levels of 84%, 77%, and 59% for three, two and one vaccine doses, respectively. Greater or lower effectiveness values would result in greater or lower levels of anti-pertussis herd immunity, respectively. However, the vaccination effectiveness assumed in this study was based on evaluative studies [20,21,22]. Fourthly, the method to determine the prevalence of vaccine-induced pertussis protection required to establish herd immunity is based on two assumptions: (1) homogeneous mixing of individuals within the population and (2) homogeneous distribution of protected individuals within the population [10,52]. If a country or WHO region does not have a homogeneous distribution of protected individuals, the prevalence required to establish herd immunity will be lower/greater in areas with greater/lower prevalences. However, the information about the distribution of protected individuals in different countries was not available. Fifthly, anti-pertussis herd immunity levels were assessed against *Bordetella pertussis* with R_o_ values ranging from 10 to 18. R_o_ values lower than 10 would result in values greater than those found in this study in different countries and WHO regions. However, this range of Ro values was obtained in studies assessing *Bordetella pertussis* transmissibility [10,23].

## 5. Conclusions

This study found that global and regional DTP vaccination coverage and anti-pertussis herd immunity indicators in 2024 did not recover completely to pre-COVID-19 levels. Eight of the ten routine DTP indicators assessed in this study worsened globally and in WHO regions from 2029 to 2024, including the mean DTP1 coverage, mean DTP3 coverage, three-dose coverage, zero-dose coverage, percentage of countries with three-dose coverage ≥ 95%, percentage of countries with three-dose coverage ≥90%, percentage of countries where all children aged one year had received at least one dose of the DTP vaccine, and the mean prevalence of children aged one year with vaccine-induced pertussis protection. However, the differences between 2019 and 2024 were statistically significant only for the three-dose DTP coverage decrease in the European region and the decrease in the global percentage of countries with ≥ 90% three-dose coverage. In 2024, the zero-dose coverage indicators assessed in this study were not on track to achieve the Immunization Agency 2030 objective. Routine DTP vaccination coverage should be increased in all WHO regions, but specially in the high-priority countries for DTP vaccination coverage identified in this study.

## Figures and Tables

**Table 1:** DTP vaccination indicators (mean %, 95% CI) worldwide and in WHO regions in 2024: DTP1 coverage; DTP3 coverage; zero DTP1-based coverage; three-dose coverage; two-dose coverage; one-dose coverage; zero-dose coverage (conservative approach); percentage of countries with three-dose DTP coverage ≥ 95 and ≥90; prevalence of one-year-old children with vaccine-induced pertussis protection; and percentage of countries with herd immunity in one-year-old children (target population) with vaccination against *Bordetella pertussis* (R_o_ from 10 to 18).

	World	African Region	Americas Region	Eastern Mediterranean Region	European Region	South-East Asia Region	Western Pacific Region
No. of countries	195	47	35	22	53	11	27
Mean (%, 95% CI) DTP1 and DTP3 routine vaccination coverage							
DTP1	90.7 (89.2–92.2)	85.5 (82.4–88.6)	89.7 (86.2–93.1)	89.0 (82.4–95.7)	94.8 (93.1–96.5)	93.7 (88.5–98.9)	93.3 (89.2–97.5)
DTP3	86.6 (84.7–88.4)	79.9 (76.3–83.6)	86.4 (82.2–90.5)	84.0 (75.5–92.6)	91.9 (89.7–94.1)	91.4 (84.9–97.8)	88.0 (82.6–93.3)
Zero-dose (100 – DTP1)	9.3 (0–28.7)	14.5 (5–24.1)	10.3 (2.1–18.6)	11.0 (4.4–17.5)	5.2 (0–15.3)	6.3 (1.5–11)	6.7 (0–13.9)
Mean (%, 95% CI) three-, two-, one- and zero-dose DTP vaccination coverage (conservative approach)							
Three-dose ^a^	72.8 (69.6–76)	59.5 (53.1–65.9)	71.0 (63.1–78.9)	71.0 (57.9–84)	82.5 (78.4–86.6)	81.1 (67.6–94.5)	77.5 (68.9–86.1)
Two-dose	21.1 (19.1–23.1)	30.4 (26.7–34)	22.7 (17.5–27.8)	19.6 (13–26.1)	15.3 (12.4–18.1)	15.8 (5.9–25.6)	17.7 (12.5–23)
One-dose	5.3 (4.1–6.4)	8.9 (6.3–11.5)	5.6 (3–8.2)	7.5 (1.8–13.2)	2.0 (0.8–3.3)	2.9 (0–6.3)	4.0 (0.6–7.3)
Zero-dose ^b^	0.8 (0.5–1.2)	1.2 (0.5–1.9)	0.7 (0.2–1.1)	1.9 (0–4)	0.2 (0–0.5)	0.3 (0–0.6)	0.5 (0–2)
Percentage (%, 95% CI) of countries with three-dose DTP vaccination coverage ≥95% and ≥90%							
≥95%	12.3 (3.9–16.9)	2.1 (0–6.3)	17.1 (4.7–29.6)	9.1 (0–21.1)	15.1 (5.5–21.1)	18.2 (0–41)	18.5 (3.9–33.2)
≥90%	21.0 (15.3–26.7)	4.2 (0–10)	20.0 (6.7–33.3)	9.1 (0–21.1)	34.0 (21.2–46.7)	18.2 (0–41)	37.0 (18.8–55.3)
Mean prevalence (%, 95% CI) of one-year-old children with vaccine-induced pertussis protection							
Pertussis immunity	80.5 (79.9–81.2)	78.6 (77.2–80)	80.4 (79.1–81.8)	79.1 (75.7–82.6)	82.3 (81.6–82.9)	82.0 (80.1–83.8)	81.1 (79.1–83.1)
Percentage of countries with sufficient herd immunity against *Bordetella pertussis* with R_o_ from 10 to 18							
R_o_ of 10–18 ^c^	0	0	0	0	0	0	0

CI: confidence interval. ^a^ Three-dose DTP coverage determined from DTP3 × DTP2 × DTP1 (conservative approach) [10]. ^b^ Zero-dose DTP coverage determined from 100 − (three-dose coverage + two-dose coverage + one-dose coverage) (conservative approach) [10]. ^c^ Pertussis transmissibility in terms of basic reproduction number R_o_. The basic reproduction number is the average number of secondary pertussis infections produced per infective case in a totally susceptible population.

**Table 2:** Variation from 2019 to 2024 for DTP vaccination indicators worldwide and in WHO regions: DTP1 coverage; DTP3 coverage; zero DTP1-based coverage; three-dose coverage; two-dose coverage; one-dose coverage; zero-dose coverage (conservative approach); percentage of countries with three-dose DTP coverage ≥ 95 and ≥90; prevalence of one-year-old children with vaccine-induced pertussis protection; and percentage of countries with herd immunity in one-year-old children (target population) with vaccination against *Bordetella pertussis* (R_o_ from 10 to 18).

	World	African Region	Americas Region	Eastern Mediterranean Region	European Region	South-East Asia Region	Western Pacific Region
No. of countries	195	47	35	22	53	11	27
Mean (%) DTP1 and DTP3 vaccination coverage							
DTP1	−2.0	−2.3	−3.4	−0.8	−2.3	−2.6	0.0
DTP3	−1.8	−1.4	−2.2	−1.1	−2.6	−2.3	−0.9
Zero-dose (100 − DTP1)	25.4	6.7	49.9	36.9	44.7	56.8	17.0
Mean (%) three-, two-, one- and zero-dose DTP vaccination coverage (conservative approach)							
Three-dose ^a^	−5.5	−7.2	−6.5	−0.4	−6.4 *	−5.6	−2.9
Two-dose	17.2 *	16.5	12.9	−6.2	37.8 *	22.5	20.4
One-dose	23.3	7.2	51.4	13.6	150.0	141.7	−7.0
Zero-dose ^b^	14.3	−20.0	133.3	58.3	-	-	−33.3
Percentage of countries with three-dose DTP vaccination coverage ≥ 95% and ≥90%							
≥95%	−31.7	−50.0	19.6	−61.8	−33.2	0.0	−44.4
≥90%	−38.2 **	−34.4	−22.2	−68.2	−35.6	−66.6	−28.6
Mean prevalence (%) of vaccine-induced pertussis protection in one-year-old children							
Pertussis immunity	−0.7	−0.4	−1.2	−0.9	−0.8	−1.0	0.2
Percentage of countries with sufficient herd immunity against *Bordetella pertussis* with R_o_ from 10 to 18							
R_o_ of 10−18 ^c^	0	0	0	0	0	0	0

CI: confidence interval, * *p* < 0.05, ** *p* < 0.005 for the comparison between 2024 and 2019. ^a^ Three-dose DTP coverage determined from DTP3 × DTP2 × DTP1 (conservative approach) [10]. ^b^ Zero-dose DTP coverage determined from 100 − (three-dose coverage + two-dose coverage + one-dose coverage) (conservative approach) [10]. ^c^ Pertussis transmissibility in terms of basic reproduction number R_o_. The basic reproduction number is the average number of secondary pertussis infections produced per infective case in a totally susceptible population.

**Table 3:** No. (%) of high-priority countries for routine DTP vaccination coverage increase based on four criteria worldwide and in different WHO regions: (1) zero-dose DTP coverage greater than the regional mean coverage; (2) three-dose DTP coverage lower than 60%; (3) DTP1 coverage lower than 80%; (4) DTP3 coverage lower than 80%.

Priority Criteria	No. (%) of High-Priority Countries for Routine DTP Vaccination Coverage Increase Based on Four Criteria						
	World	African Region	Americas Region	Eastern Mediterranean Region	European Region	South-East Asia Region	Western Pacific Region
0-dose DTP coverage > regional mean	37 (19.0)	15 (31.9)	7 (20.0)	5 (22.7)	4 (7.5)	2 (18.2)	4 (14.8)
3-dose DTP Vaccination + coverage < 60%	48 (24.6)	22 (46.8)	12 (34.3)	6 (27.3)	3 (5.7)	1 (9.1)	4 (14.8)
DTP1 vaccination coverage < 80%	27 (13.8)	13 (27.7)	6 (17.1)	3 (13.6)	2 (3.8)	1 (9.1)	2 (7.4)
DTP3 vaccination coverage < 80%	47 (24.1)	21 (44.7)	9 (25.7)	6 (27.3)	1 (5.7)	2 (18.2)	6 (22.2)
No. of countries	195	47	35	22	53	11	27

## Data Availability

The original contributions presented in this study are included in the article. Further inquiries can be directed to the corresponding author.

## Supplementary Materials

The following supporting information can be downloaded at: https://www.mdpi.com/article/10.3390/vaccines14030264/s1, Table S1. DTP vaccination indicators. (mean %, 95% CI) worldwide and in WHO regions in 2019: DTP1 coverage; DTP3 coverage; zero DTP1-based coverage; three-dose coverage; two-dose coverage; one-dose coverage; zero-dose coverage (conservative approach); percentage of countries with three-dose DTP coverage ≥ 95 and ≥90; prevalence of one-year-old children with vaccine-induced pertussis protection; and percentage of countries with herd immunity in one-year-old children (target population) with vaccination against *Bordetella pertussis* (R_o_ from 10 to 18). Table S2: High-priority countries for DTP vaccination coverage increase in different WHO regions, based on four indicators: (1) zero-dose DTP coverage lower than the regional mean (very high priority); (2) three-dose DTP coverage < 60%; (3) DTP1 vaccination coverage < 80%; and (4) DTP3 vaccination coverage < 80%.
