# Supplementary material for: Routine DTP Vaccination Coverage and Herd Immunity Against Pertussis in 2024 Did Not Recover to Pre-COVID-19 Levels Globally and in WHO Regions
Source: Vaccines (Basel). 2026 Mar 13;14(3):264. doi: 10.3390/vaccines14030264 (PMC13030230; doi:10.3390/vaccines14030264)
Supplement: Supplementary file 1 [file vaccines-14-00264-s001.zip › vaccines-4149204-supplementary.pdf]

Supplementary Materials

Table S1. DTP vaccination indicators.(mean %, 95% CI) worldwide and in WHO regions in 2019: DTP1 coverage; DTP3 coverage; zero DTP1-based coverage; three-dose coverage; two-dose coverage; one-dose coverage; zero-dose coverage (conservative approach); percentage of countries with three-dose DTP coverage ≥95 and ≥90; prevalence of one-year-old children with vaccine-induced pertussis protection; and percentage of countries with herd immunity in one-year-old children (target population) with vaccination against Bordetella pertussis (R_o from 10 to 18)

	World	African Region	American Region	Eastern Mediterranean Region	European Region	South-East Asia Region	Western Pacific Region
No. of countries	194	47	35	21	53	11	27
DTP1	92.6	87.5	92.8	89.8	97	96.2	93.3
DTP3	88.2	81.1	88.3	85	94.4	93.5	88.8
Zero-dose 100 − DTP1	7.4	13.6	6.9	8.0	3.6	4.0	5.7
Mean (%) three-, two-, one- and zero-dose DTP vaccination coverage (conservative approach)							
Three-dose[a]	77.0	64.1	75.9	71.3	88.1	85.9	79.8
Two-dose	18.0	26.1	20.1	20.9	11.1	12.9	14.7
One-dose	4.3	8.3	3.7	6.6	0.8	1.2	4.3
Zero-dose[b]	0.7	1.5	0.3	1.2	0.0	0.0	1.2
Mean prevalence (%) of vaccine-induced pertussis protection in one-year-old children							
Pertussis immunity	88.1	77.6	91.8	88.2	93.7	93.2	88.8
Percentage of countries with three-dose DTP vaccination coverage ≥ 95% and ≥90%							
≥95%	18.0	4.2	14.3	23.8	22.6	18.2	33.3
≥90%	34.0	6.4	25.7	28.6	52.8	54.5	51.8
Percentage of countries with herd immunity established against pertussis viruses with Ro from 10 to ≥18							
Ro of 10–18[c]	0	0	0	0	0	0	0

a. Three-dose DTP coverage determined from: DTP3 x DTP2 x DTP1 (conservative approach) [10].
b. Zero-dose DTP coverage determined from: 100 − (three-dose coverage + two-dose coverage + one-dose coverage) (conservative approach) [10].
c. Pertussis transmissibility in terms of basic reproduction number R_o. The basic reproduction number is the average number of secondary pertussis infections produced per infective case in a totally susceptible population.

Table S2. High-priority countries for DTP vaccination coverage increase in different WHO regions, based on four indicators: 1) zero-dose DTP coverage lower than the regional mean; 2) three-dose DTP coverage <60%; 3) DTP1 vaccination coverage <80%; and 4) DTP3 vaccination coverage <80%.

High-priority countries for DTP vaccination coverage increase in different WHO regions

Zero-dose DTP coverage > Regional mean	Three-dose DTP Coverage < 60%	DTP1 vaccination coverage <80%	DTP3 vaccination coverage <80%
AFRICAN REGION			
Central African Republic	Central African Republic	Central African Republic	Central African Republic
Gabon	Gabon	Gabon	Madagascar
Madagascar	Madagascar	Madagascar	Gabon
Angola	Angola	Angola	Benin
Nigeria	Nigeria	Nigeria	Guinea
Benin	Benin	Guinea-Bissau	Angola
Guinea	Guinea	Equatorial Guinea	De. Rep. Congo
Guinea-Bissau	Guinea-Bissau	Benin	Nigeria
Equatorial Guinea	Equatorial Guinea	South Sudan	Guinea-Bissau
Dem. Rep. Congo	Congo	South Africa	Equatorial Guinea
Somalia	Somalia	Guinea	Chad

South Sudan	South Sudan	Somalia	Somalia
South Africa	South Africa	Namibia	Mozambique
Chad	Chad		South Sudan
Namibia	Namibia		Ethiopia
	Ethiopia		South Africa
	Comoros		Namibia
	Cote d'Ivoire		Comoros
	Cameroon		Cote d'Ivoire
	Mozambique		Cameroon
	Rep. Dem. Congo		Congo
	Gambia		
AMERICAS REGION			
Bolivia	Bolivia	Bolivia	Bolivia
Venezuela	Venezuela	Venezuela	Haiti
Haiti	Haiti	Ecuador	Venezuela
Ecuador	Suriname	Suriname	Suriname
Suriname	Ecuador	Honduras	Ecuador
Honduras	Honduras	Haiti	Panama
Argentina	Argentina		Honduras
	Mexico		Argentina
	Paraguay		Mexico
	Panama		
	Peru		
	Guatemala		
EASTERN MEDITERRANEAN REGION			
Sudan	Sudan	Sudan	Sudan
Yemen	Yemen	Yemen	Yemen
Afghanistan	Afghanistan	Afghanistan	Lebanon
Lebanon	Lebanon		Afghanistan
Syrian Arab Republic	Syrian Arab Republic		Syrian Arab Republic
	Djibouti		Djibouti
EUROPEAN REGION			
Azerbaijan	Azerbaijan	Azerbaijan	Azerbaijan
Romania	Romania	Romania	
Estonia	Estonia		
SOUTH-EAST ASIA REGION			
Myanmar	Myanmar	Myanmar	Myanmar
Indonesia			Indonesia
WESTERN PACIFIC REGION			
Papua New Guinea	Papua New Guinea	Papua New Guinea	Papua New Guinea
Lao People's Democratic Republic	Lao People's Democratic Republic	Lao People's Democratic Republic	Lao People's Democratic Republic
Philippines	Philippines		Philippines
Vanuatu	Vanuatu		Vanuatu

Cook Islands

Micronesia
